# Intention-to-treat. What is the question?

**DOI:** 10.1186/1743-7075-6-1

**Published:** 2009-01-09

**Authors:** Richard D Feinman

**Affiliations:** 1 Department of Biochemistry, SUNY Downstate Medical Center, Brooklyn, NY 11203, USA

## Abstract

It has become commonplace for Randomized Controlled Trials (RCTs) to be analyzed according to Intention-to-Treat (ITT) principles in which data from all subjects are used regardless of the subjects' adherence to protocol. While ITT analyses can provide useful information in some cases, they do not answer the question that motivates many RCTs, namely, whether the treatments differ in efficacy. ITT tends to reduce information by combining two questions, whether the intervention is effective and whether, as implemented, it has good compliance. Because these questions may be separate there is a risk of misuse. Two examples are presented that demonstrate this potential for abuse: a study on the effectiveness of vitamin E in reducing cardiovascular risk and comparisons of low fat and low carbohydrate diets. In the first case, a treatment that is demonstrably effective is described as without merit. In the second, ITT describes as the same, two diets that actually have different outcomes. These misuses of ITT are not atypical and are not technical problems in statistics but have real consequences for scientific principles and health recommendations. ITT analyses may answer the question of what happens when treatments are recommended but are inappropriate where separate information on adherence and performance is available. It is proposed that results of RCTs, or any experimental study, be reported, not in terms of the analyses that were performed, but rather in terms of the questions that the analyses can answer properly.

## Background

Randomized Controlled Trials (RCT) are typically performed to compare the efficacy of two or more treatments. One problem that has vexed investigators for as long as there have been comparative trials is that experimental subjects don't always follow instructions; in the Women's Health Intiative (WHI), for example, after 6 years, only 14% of the women randomized to cutting fat to 20% of calories were meeting their goal [1,2]. More important, in some studies, subjects drop out altogether. In the latter case, at least, it seems reasonable that data on such non-participants should not be included in the final analysis of the data.

Recently, however, a statistical concept, Intention-to-Treat (ITT) analysis, has appeared. In ITT, the data from all subjects who are randomized to treatment are analyzed regardless of whether subjects followed the protocol or not ("analyze as randomized"). At first hearing, the idea of ITT is counter-intuitive if not completely irrational – why would you include in your data, people who are not in the experiment? – suggesting that a substantial burden of proof rests with those who want to employ it. No such obligation is usually met and, particularly in nutrition studies, such as comparisons of isocaloric weight loss diets, ITT is frequently used without justification. ITT analyses are typically reported in a way that implies that they have the final say on efficacy and it is even argued that, once assigned to an experimental group, all data *must *be included in the analysis even if subjects do not comply with the protocol [3]. Dallal has described the history succinctly and summarized the arguments, pro and con in an engaging manner although ultimately coming out generally against the practice [4]. Not surprisingly, there is a good deal of controversy on this subject and because an ITT analysis can draw the exact opposite conclusion to a traditional one, the potential for confusion for the public and scientific researchers alike is great.

## What is the question?

"Unfortunately, people generally ignore the connections between the formal notions of statistics and the informal understandings and stories from which they grow. They consider numbers as coming from a different realm than narratives and not as distillations, complements or summaries of them. People often cite statistics in bald form, without the supporting story and context needed to give them meaning."

- John Allen Paulos, *Once Upon a Number. The Hidden Mathematical Logic of Stories *(ref. [5]), p. 12

Both sides of the ITT controversy have strong opinions and it is not clear how the dialogue can proceed. Here it is suggested that, in any analysis of scientific data, attention be paid to precisely what question is being asked and, regardless of labels, results be reported as answers to those questions. Thus, in a simple experiment in which the efficacy of a dietary supplement is being studied, one sensibly wants to answer the question "What happens if you take the supplement?" In other words, one is generally interested in the efficacy of the supplement. Adherence, because it may depend on the particular implementation and the behavior of the subject, is a separate question. In a more complicated experiment such as a weight loss diet comparison, a slightly different question is "can people stay on one or another diet?" ITT, because it is really asking the question "What happens if you are assigned to a particular diet?" is collapsing the two questions of adherence and efficacy into a single question. Reducing the two questions to a single one limits the information that is available. If ITT *is *used, it is important to be precise about what question is being asked. Of course, many treatments are inherently inconvenient and have unavoidable side effects, causing subjects to become non-adherent when benefits are uncertain or not immediately felt, but this cannot be *assumed *to always be the case. Whatever the general value of ITT, it is the confusion over what question has been asked that leads to widespread misuse.

The major point of this commentary is that if ITT is used, it must be clearly stated that it it is taking assignment to a protocol as the independent variable, that is, it is asking the question "what is the effect of being *assigned *to a particular group?"

In this commentary two cases are considered where inappropriate use of ITT can compromise scientific understanding and clinical recommendations: the effectiveness of use of vitamin E as a dietary supplement and the relative efficacy of low carbohydrate and low fat diets. It is argued that common sense suggests that the wrong questions were asked, or, when correctly stated, misinterpreted as a consequence of use of ITT. Although restricted to dietary comparisons, the ideas are generalizable, and an example is given of choice of therapy in coronary heart disease.

## The effect of vitamin E supplementation

A clear cut case of incorrect use of ITT is a recent report on the value of antioxidant supplements [6]. The Abstract of the paper concluded that "there were no overall effects of ascorbic acid, vitamin E, or beta carotene on cardiovascular events among women at high risk for CVD." The study, however, provided an ITT analysis and on the fourth page of the paper, it turns out that removing subjects due to "noncompliance led to a significant 13% reduction in the combined end point of CVD morbidity and mortality...with a 22% reduction in MI ..., a 27% reduction in stroke .... a 23% reduction in the combination of MI, stroke, or CVD death (RR (risk ratio), 0.77; 95% CI, 0.64–0.92 [*P *= .005])." The media universally reported the conclusion from the Abstract, namely that there was no effect of vitamin E. This conclusion is incorrect if precise language is used. No effect was the answer to the question about the outcome of the assignments. It is reasonable that the effect of the vitamins means the effect of taking the vitamin and the data do show a significant effect. From the standpoint of public health and preventive medicine, it is hard to see that ITT was the right analysis.

## "The same at one year"

A more subtle problem of asking the right question is the study of macronutrient composition of diets. The question is of current interest in that the American Diabetes Association (ADA), long an opponent of the use of low carbohydrate diets (< 130 g/day), for the first time in 2008, gave weak endorsement to such diets for weight loss [7]. While the change in position was ascribed to evolving research, the data referenced are two studies from 2003 and 2004 comparing low-carbohydrate and conventional diets. The studies of Stern, et al. [8] and of Foster, et al. [9] are widely cited as showing that weight loss is better on a low-carbohydrate diet at 6 months but is the same as that for a low fat diet at 1 year and this is the interpretation accepted by the ADA. It has, however, been pointed out that because the low carb diet allowed re-introduction of carbohydrate as the experiment proceeded, the diets became similar at one year and that the implication was that increased carbohydrate reduced effectiveness [10,11]. In any case, the idea that low-fat and low-carbohydrate diets give the same results at one year persists and has been quoted numerous times in the literature. Analysis of the data, however, suggests that it is not correct.

Stern, et al. [8] are apparently up front about the study and report in their Abstract: "Participants received counseling to either restrict carbohydrate intake... or to restrict caloric intake." However, the Results in the Abstract are reported: "By 1 year, mean (± SD) weight change for persons on the low-carbohydrate diet was -5.1 ± 8.7 kg compared with -3.1 ± 8.4 kg for persons on the conventional diet. Differences between groups were not significant (-1.9 kg [95% CI, -4.9 to 1.0 kg]; P = 0.20) .... Conclusion: ... Weight loss was similar between groups [at 1 year]...."

It is reasonable to assume, however, that persons described as being on a diet have stayed on the diet, unless it is clearly explained that they have not. Of course, compliance to a long term diet may be very variable and, as in the WHI [1], compliers may not all have had the same nutritional intake. Nonetheless, it was possible to identify those who had dropped out of the study and as part of the analysis, the authors explain in the text ([8], p. 781) that the data included the results for those who had dropped out. When this sub-group is examined it was found that: "Persons on the low-carbohydrate diet who dropped out lost less weight than those who completed the study (change, -0.2 ± 7.6 kg vs. -7.3 ± 8.3 kg, respectively; mean difference, -7.1 kg [CI, -11.6 kg to -2.8 kg]; *P *= 0.003)." This result is not surprising. Reflection on the statement, however, suggests that the counter-intuitive idea that you can be considered to be on a diet even if you drop out, has been tacitly assumed.

The report continues, "In contrast, weight loss was *not significantly different for those on the conventional diet, whether they dropped out or completed the study *(change, -2.2 ± 9.5 kg vs. -3.7 ± 7.7, respectively; mean difference, -1.5 kg [CI, -5.7 kg to 2.7 kg]; *P *> 0.2). Nevertheless, the difference in weight loss between the 2 diet groups for those who dropped out of the study was not significant (*P *>0.2)." (My italics).

The last two sentences mean that following a conventional diet is indistinguishable from *not *following such a diet. In other words, it doesn't matter whether you follow a conventional diet or merely say you are going to follow a conventional diet. To understand how this astounding conclusion could be taken as evidence for the equality of the two diets, one must consider what question is being asked. Although, as noted above, the Abstract of Stern, et al. begins circumspectly, the question shifts from the effect of assignment to the diet to a question about the diet itself. The inclusion of non-compliers is hidden, that is, one does not know, until the body of the paper, that some of the participants did not participate. The question about assignment is different from the question of efficacy unless it is assumed that adherence is an unalterable feature of the diet, rather than an effect of patient motivation and experimental protocol. In practice, dieters rely on all sorts of stimuli that may or may not be in the experimental protocols. A survey [12] of the Active Low-Carber Forums [13], an on-line support group showed that there was variable reliance on information from different sources (e.g. popular books, TV or other media, manufacturers websites, online support forums) which can be separately controlled by different protocols. When the question of adherence is separated from efficacy, the correct conclusion about the study of Stern, et al. is: Weight loss was **not** similar between groups at 1 year (Table 1).

**Table 1:** Weight Loss in Diet Comparisons and the Effect of Analysis. Data from References [8,9].

		Data for 12 months Weight Loss (kg)			
		With Drop-outs	SD	Only Study Subjects	SD

Foster, et al.	low carb	4.4	6.7	7.3	7.3
	low fat	2.5	6.3	4.5	7.9
	difference	**1.9**		**2.8**	

Stern, et al.	low carb	5.1	8.7	7.3	8.3
	low fat	3.1	8.4	3.7	7.7
	difference	**2**		**3.6**	

The study by Foster, et al. [9], which might be said to have sparked the recent low carbohydrate revolution provided similar data (Table 1): the difference between an ITT analysis and one based separately on performance of compliers is clearly substantial. Whether the difference in results is significant is not stated but it would be hard to say that the diets are the same.

Thus, the conclusion that weight loss is the same at 1 year on low-carbohydrate diets and conventional diets comes from an ITT analysis and, as stated, is misleading. Because dieters and practitioners reasonably want to know the potential of a diet, it seems that authors must be very circumspect about describing results. The ITT analysis, again, only answers the question about assignment to a diet in a particular experimental setting, and does not address the question as to which is the more effective diet if adhered to. The fact that it is acknowledged that the substantially greater improvement in plasma triglycerides on the low carbohydrate diet compared to the low fat diets persisted for one year should have been taken as a sign that it would be surprising if the diets were the same [7,9].

## Common sense argument against intention-to-treat

Consider an experimental comparison of two diets in which there is a discrete outcome, e.g. a threshold amount of weight lost or remission of an identifiable symptom. (The analysis is easily generalized to a continuous outcome or to a drug trial.) Patients are randomly assigned to two different diet, diet group A or diet group B and a target of 5 kg weight loss is considered success. Assume that half of the subjects in group A are able to stay on the diet and half are not. The patients in this group who stayed on the diet, however, are all able to lose the target 5 kg. Now in group B, imagine that everybody is able to stay on the diet but only half are able to lose the required amount of weight. An ITT analysis shows no difference in the two outcomes. With such data in hand should a physician advise a patient: "well, the diets are pretty much the same. It's largely up to you which you choose," or, looking at the raw data (both compliance and success), should the recommendation be: "Diet A is much more effective than diet B but people have trouble staying on it. If you can stay on diet A it will be much better for you so I would encourage you to see if you could find a way to do so." Which makes more sense?

Although diet trials are emphasized here, it is worthwhile to apply the common sense test to a study where the authors insist that ITT is appropriate. In the Coronary Artery Bypass Surgery (CABS) trial, reproduced by Newell [3], patients were assigned to Medicine or Surgery. The actual modalities used and the outcomes are shown in Table 2. Intention-to-treat analysis was, as described by Newell, "used, correctly." A 7.8% mortality was found in those allocated to medical treatment, and a 5.3% mortality for assignment to surgery. Values for those who actually received the assigned treatment were 8.4% for medicine, and 4.1% for surgery. If, however, we look at the outcomes of each modality as actually implemented, it turns out that that medical treatment had a 9.5% (33/349) mortality rate compared with 4.1% (17/419; P = 0.003) for surgery, an analysis that Newell says "would have wildly exaggerated the apparent value of surgery." Common sense, however, suggests that appearances are not deceiving and patients and physicians should try as hard as possible to implement surgery. Common sense dictates that a patient is interested in surgery, not the effect of being assigned to surgery, and that a patient has a right to expect that if they comply, the physician would avoid conditions where, as stated by Hollis [14] "most types of deviations from protocol would continue to occur in routine practice." The idea that "Intention to treat analysis is ... most suitable for pragmatic trials of effectiveness rather than for explanatory investigations of efficacy" assumes again that practical considerations are the same everywhere and that any practitioner is locked into the same abilities as the experimenter.

**Table 2:** Survivors and deaths, 2 years after allocation to surgery or medical treatment in the CABS trial. Data as reported in reference [3]

	Allocated to medicine		Allocated to surgery	
	
	Received surgery	Received medicine	Received surgery	Received medicine
Survived 2 years	48	296	354	20

Died	2	27	15	6

Total	50	323	369	26

## Geometric argument against ITT

What is actually accomplished in an ITT analysis? A dietary intervention (or drug trial) has, in the simplest case, two outcomes, adherence and efficacy. A geometrical argument would describe the results of the experiment as a 2-dimensional outcome space where the length of a vector tells how every subject did but the separate coordinates of outcome and efficacy would be reported. ITT represents a projection of the vector onto one axis, in other words collapses a two dimensional vector to a one-dimensional vector, thereby losing part of the information.

## Bias

The supposed value of ITT is that "an analysis that excludes noncompliant patients is no longer randomized and might cause serious bias" but "bias" is an emotionally charged word and it is not clear what kind of bias is introduced. In fact, the kind of misleading bias that is implied in the term is what happens when you leave non-compliers in the study. Consider that if it were known before the experiment that some subjects, for whatever reason, religious or psychological, would refuse to take the medication or would refuse to consume the designated diet, would it not make sense to exclude them in the trial for fear that the results would be biased. Finding out after the fact that they were different from your intended study population does not change anything; *including *them introduce *s *bias. Hollis [14] describes how this bias could be avoided "by randomization after the necessary event, but this is not always possible in practice." It is not clear why not. It is in fact what is done by discarding non-compliers, and what is done in any scientific experiment in which the data rather than the design of the experiment are paramount.

## Appropriate Uses of Intention to Treat

The most salient characteristic of the epidemic of obesity and diabetes, is that the increase in caloric intake is almost entirely due to carbohydrates. Government databases show that the absolute amount of dietary fat stayed roughly the same, or actually decreased for men and only slightly increased for women: per cent of dietary fat and saturated fat, of course, went down [15,16]. This might well be described as a vast thirty year experiment in which subjects were advised to reduce dietary fat and increase carbohydrate – the previous thirty years might be considered a control group. The null hypothesis is that being assigned to a population advised to reduce dietary fat would have no improvement or would make worse obesity, heart disease and general health. These are true ITT conditions: we do not know if the poor outcome was due to the group that did not follow the protocol and that those who did reduce fat actually got better but could not overpower the statistics. Moreover, we do not know the mechanism and it is said the obesity epidemic is due to reduced exercise and larger portion sizes. It is possible that carbohydrate intake leads one to consume larger portions or that the soporific effect of high carbohydrate intake leads to lethargy but an ITT analysis simply says that being assigned to a low fat group correlates with obesity and diabetes and presumably incidence of heart disease (the data are not unambiguous here but it would be surprising if the incidence of heart disease went down in an increasingly obese and diabetic population).

A similar analysis applies to the recent results of the Women's Health Initiative (WHI) where, as described by the authors "over a mean of 8.1 years, a dietary intervention that reduced total fat intake and increased intakes of vegetables, fruits, and grains did not significantly reduce the risk of CHD, stroke, or CVD..." and only a limited weight loss compared to controls [1]. An ITT analysis, which is all that one can do since individual data was not reported, says that the null hypothesis is correct: the intervention as individuals were randomly assigned had little effect. It is important to point out, however, that, in the WHI, it is not just that the assignment to the intervention had little effect. There *was *a change in the behavior of the experimental group who did reduce the amount of fat (although not to the target 20%) and did increase the amount of fruits and vegetables, which is why the results were generally considered disappointing. An ITT analysis says that actually reducing fat does not have the expected beneficial outcome. The question that readers would most want answered is not "what is the effect of being assigned to the diet?" or "what is the effect of trying to comply with the diet" but rather, as stated by the authors, what is the effect of "reduced total fat intake and increased intakes of vegetables, fruits, and grains?"

## Summary

The ITT approach has been advanced as a way to overcome the difficulties faced by a per protocol analysis when answering the question of efficacy. It avoids the problem of differential drop out when the likelihood of a subject's becoming non-adherent is related to treatment and outcome. For example, if subjects who did not lose weight during a diet study were to drop out, all diets would appear to be equally effective. An ITT analysis would include the weight changes from non-adherent subjects. ITT analyses are typically reported in a way that implies that they have the final say on efficacy; Newell, for example [3] insists that "In any study of a health intervention, it is *essential *to remember patients or clients who would not or could not complete the planned intervention, and include them appropriately in the analysis." (My italics).

An ITT approach, because it includes non-adherent subjects, cannot give the definitive answer as to how treatments compare in adherent subjects. The ITT approach answers a different question, namely, what happens when people are assigned to a particular treatment or what happens when a particular treatment is recommended. Again, if authors choose to report that, they must make clear what question is being asked.

The possibility that the questions of compliance and efficacy are not independent – that, for example, a diet that worked well might encourage dieters' compliance, while reasonable in many cases, needs to be proved and cannot be assumed at the experimenter's will. In some sense, the name tells the story. All kinds of complicated experiments are paved with good intentions, but it is the experiment not the experimenter that counts.

## Practical consequences and recommendations

The ITT approach has gained ascendancy in the world of medical research and may actually be required by some editors or granting agencies (see Supplemental Material). Researchers may thus have no choice but to perform such an analysis but it should not be done without awareness of what question is being asked, and whether it is, in fact, the one that is most desirable. Analyses that separate adherence and efficacy may, in fact, be more relevant. This is especially true if we want to make health recommendations. Marantz [17] has stated the principle well: "Public health, just like personal-encounter medicine, should be guided by the dictum "first, do no harm." Sometimes, in the absence of clear and convincing evidence of net benefit, that will mean: do not issue dietary guidelines at all." Whether vitamin E should be recommended is open to further study but should not be dismissed on the basis of a faulty analysis. Similarly, it may well be that the evidence that carbohydrate-restricted diets are better than other nutritional approaches is insufficient to warrant blanket recommendation but that evidence should not be compromised by an unjustifiable statistical principle. Unless one assumes that compliance is an unalterable character trait and that health professionals cannot effectively encourage dieters at all, one might be concerned about negative impact of people ignoring a diet or supplement that might be effective. These problems are not a statistical niceties and have the potential for practical harm and should be considered carefully.

Authors my choose to use both ITT and the performance of compliers but, again, if ITT is used, it must be clearly stated that it is taking assignment to a protocol as the independent variable, that is, it is asking the question "what is the effect of being *assigned *to a particular group?"

## The ITT controversy

Advocates of ITT see its principles as established and may dismiss a common sense approach as naïve. The issue is not easily resolved; statistics does not truly derive from *a priori *principles. Most statisticians would agree that the discipline, in general, is a way of quantifying our intuitions. If this is not appreciated, and one does not go back to the question to be asked, it is easy to develop a dogmatic approach and insist on a particular statistic because it has become standard. An anonymous reviewer of an earlier version of this paper suggested that "the arguments presented by the author may have applied, maybe, ten or fifteen years ago." This criticism reminds one of Molière's *Doctor in Spite of Himself*:

Sganarelle is disguised as a doctor and spouts medical double-talk with phony Latin, Greek and Hebrew to impress the client Geronte who is pretty dumb and mostly falls for it but:

*Geronte*: ...there is only one thing that bothers me: the location of the liver and the heart. It seemed to me that you had them in the wrong place: the heart is on the left side but the liver is on the right side.

*Sgnarelle*: Yes. That used to be true but we have changed all that and medicine uses an entirely new approach.

*Geronte*: I didn't know that and I beg your pardon for my ignorance.

In the end, it is reasonable that scientific knowledge be based on real observations. This has never before been thought to include data that was not actually in the experiment. I doubt that *nous avons changé tout cela*.
